# Native Cyclodextrin-Based Metal–Organic Frameworks (MOFs): Synthesis, Characterization, and Potential Applications in Food Industry

**DOI:** 10.3390/molecules30020293

**Published:** 2025-01-13

**Authors:** Siddanth Saxena, Manuel J. Lis

**Affiliations:** INTEXTER-UPC, Surface Science Laboratory, Colon 15, 08222 Terrassa, Spain; siddanth.saxena@upc.edu

**Keywords:** cyclodextrin, metal organic frameworks, food applications

## Abstract

Metal–organic frameworks (MOFs) have become a highly usable system in various sectors because of their highly ordered structure and high porosity providing them with high storage capacity. However, their use is sometimes forbidden in the food industry due to the presence of some organic compounds which have undesirable effects. Cyclodextrins, which are considered GRAS (Generally Recognized as Safe) by the FDA, comes as a very good alternative to previously used compounds for the development of the MOFs to be used in the food packaging industry, especially in the packaging sector. The cyclodextrin MOF does possess edible, biocompatible, as well as biodegradable characteristics and due to these reasons, they have gained attention from researchers in the food industry. In this review, we focus on the recent advancements in the field of CD MOFs. We have emphasized the synthesis of these MOFs through different techniques, formations of their inclusion complex with bioactive compounds, and their characterization. Finally, we discussed the use of CD MOFs as carriers for various highly volatile bioactive compounds and their ability to increase the solubility and stability of these bioactive compounds.

## 1. Introduction

Metal–organic frameworks are crystalline hybrid porous network materials formed by the self-assembly of inorganic metal ions/nodes and organic bridging ligands [1]. These coordination polymers have a high surface area, and their shape and size can be controlled and modified [2,3,4]. Metal–organic frameworks possess versatile applications such as gas storage and adsorption [5,6], catalysis, electrochemistry, drug delivery, and sensors molecular recognition. MOFs based on cyclodextrins (CDs) have been used in pharmaceuticals, but in recent years, CD MOFs have received attention as a potential candidate to be used in various food applications because of the biocompatibility and nontoxic nature of CDs. However, only biofriendly and biocompatible metal ions such as K^+^, Ca^2+^, and Ti^4+^ can be used as these are considered to be nontoxic in nature and can be used in food industry within acceptable ranges [7].

Cyclodextrins are cyclic oligosaccharides composed of glucose monomeric units which are linked through alpha 1-4 glycosidic linkages [8,9,10]. CDs are produced as a result of an intramolecular transglycosylation (cyclization) reaction during the degradation of starch by the CGTase enzyme [9,10]. The three types of cyclodextrins that are naturally obtained are α (hexasaccharide), β (heptasaccharide), and γ (octasaccharide). The nature of the enzyme used while production decides the distribution of α-, β-, and γ-CDs [10]. CDs possess truncated cone structures with their outer surface being hydrophilic while the inner cavity is lipophilic in nature. The cavity, lined with non-polar groups like C3, O4 glycosidic bond (ether-like), and C5–CH (aliphatic-like) groups attributed to its hydrophobicity [11]. Their hydrophobic cavity acts as an excipient for various active molecules to form complexes in order to achieve better solubilization, better physical and chemical stability, and better delivery [12]. The diameter for a α-, β-, and γ-CDs range from 4.7 to 5.3, 6.0 to 6.5, and 7.5 to 8.3 Å, respectively, which shows the difference in their suitability to form an inclusion complex with compounds based on their molecular size. β-CD is stated as the most suitable molecule due to its higher ability to form an inclusion complex with food-based additives, but due to its O2-O3 intramolecular hydrogen bond it has a very low solubility in water [13], while its counterparts α- and γ-CDs possess higher water solubility because in α-cyclodextrin the hydrogen bond belt is incomplete because of the distorted position of one glucose unit in the structure, whereas γ-cyclodextrins have a non-coplanar structure that makes them the most soluble of the native cyclodextrins [10]. CDs with larger cavity sizes also exist but both their production is difficult and they possess limited ability to form inclusion complexes [14]. Table 1 compares the different properties of the three native CDs [15].

**Figure 1:**
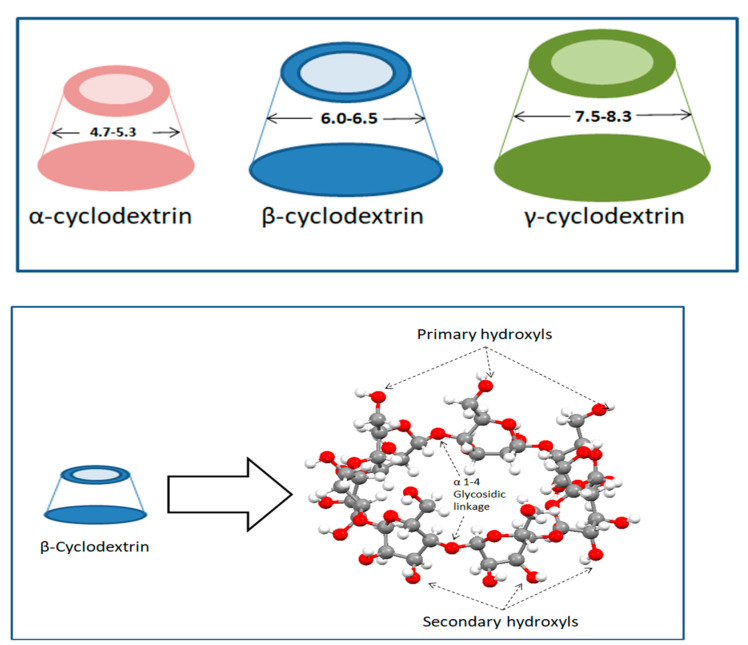
General structure of cyclodextrin (**top**). View into the molecular structure of β-cyclodextrin (**bottom**).

As discussed above, the three naturally occurring cyclodextrins namely α-, β-, and γ-CDs possess a hydrophobic inner cavity. In aqueous solution, the CD cavity is filled with the water molecules [16]. These water molecules are not able to complete the hydrogen bonding network as in bulk medium and are energetically frustrated [11,17]. These frustrated water molecules facilitate the inclusion of guest molecules through their replacement by the less polar molecule, thus decreasing the strain on the cyclodextrin ring, resulting in a more stable, lower energy state [10,16,18]. Due to their cyclic structure, CDs are less susceptible towards enzymatic degradation than the linear dextrins and CDs are better complexing agents and solubilizers [14] (see Figure 1).

**Figure 2:**
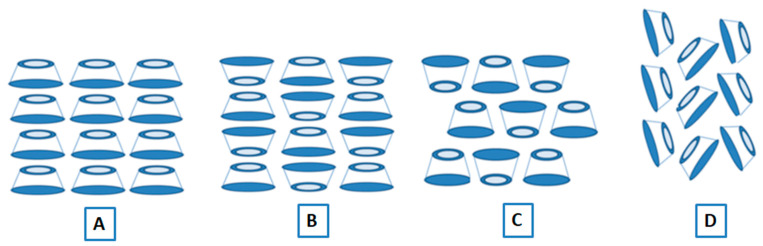
Crystal structure of cyclodextrins: (**A**) channel structure (head to tail), (**B**) channel structure (mixed), (**C**) cage structure (Brick-type), (**D**) cage structure (Herringbone).

Native cyclodextrins and their inclusion complexes exist in two crystal structures: channels and cages [13,19]. In the channel type, the molecules are placed exactly on top of each other, which creates a channel due to the alignment of their cavities in symmetry. The stacking of the cyclodextrin units can be head to head or head to tail depending on various factors. In the case of the cage-type crystal, the stacking of the CD units is such that all the cavities are blocked by each other; therefore, the guest molecules remain isolated. The packing in a cage-type crystal structure depends on the complexed guest compound and can be of two types: “Herringbone” and “Brick-type” (see Figure 2).

Though previously there have been reviews reporting similar kind of information, this review aimed to bring all the information starting from knowledge about cyclodextrin molecules to its conversion into metal–organic frameworks and further application in the food industry under one umbrella. The uniqueness of this review lies in providing information not just about food applications but also about cyclodextrin MOFs, understanding the effect of different metal anion and cations on the structure. The review is framed to provide detailed information about the structure of cyclodextrin MOFs and their preservative effect on food products. We have also emphasized on the characterization of CD-MOFs and CD-MOF inclusion complexes which provide necessary information about the techniques the researchers need to use in order to use CD-MOFs in food-based applications. We believe that this work provides complete information for future research in this field.

## 2. Toxicological Profile of Cyclodextrins

The basic concern regarding the use of cyclodextrin in food-based applications is its toxicity. The toxicity of cyclodextrins depends on the route of administration [20,21,22]. α and β CDs are resistant to β-amylases that hydrolyze starch from the non-reducing end, but they are slowly hydrolyzed by α-amylases that hydrolyze starch from within the carbohydrate chain. The only CD that α-amylase is readily hydrolyzes is the unsubstituted γ-CD [23,24,25]. Cyclodextrins are either not or only partially absorbable in the human gastrointestinal tract [26,27,28]. Undigested cyclodextrins can be metabolized by microbiota in the lower part of the gastrointestinal tract [27,28,29] after oral administration; γ-CD is completely digested in the gastrointestinal tract, whereas αCD and βCD, as well as the CD derivatives, are predominantly digested by bacteria in the colon. α-CD is digested more slowly than βCD. After parenteral administration, CDs are mainly (>90%) excreted unchanged in the urine via glomerular filtration, with the rest eliminated by other excretion pathways, such as liver metabolism and biliary excretion [29]. When the administration is oral, CDs are practically nontoxic because of their hydrophilic and bulky nature [22]. The bioavailability of natural CDs and some derivatives is also very low, making them safe when administered orally [27,29].

In the USA, they have obtained the GRAS (Generally Recognized as Safe) status as approved by the FDA (US Food and Drug Administration). In Europe, they are considered food additives and labelled as E-457 (αCDs), E-458 (γ-CDs), and E-459 (β-CDs) [30,31]. The FAO/WHO (Food and Agriculture Organization of the United Nations/World Health Organization) Joint Expert Committee on Food Additives (JECFA) set the maximum advisable level of β-CD in food at 5 mg/kg of body weight per day; however, α-CD and γ-CD do not have an established Acceptable Daily Intake (ADI) due to their benign toxicological profiles [27]. Current regulations suggest that the migration of cyclodextrins from packaging to food does not have harmful effects on consumers if they are part of the packaging; this is why cyclodextrins are recognized as safe additives for use in food [15,27].

## 3. Synthesis of Cyclodextrin MOFs

Various techniques for the synthesis of CD MOFs like V Vapor diffusion, solvothermal, hydrothermal, microwave mediated, and ultrasound mediated have been reported in the literature, but the most favorable technique in applications pertaining to food industry is the V Vapor diffusion method.

The vapor diffusion method proves to be advantageous as it provides crystal formation with high yield along with the advantage of being easy to set up and control. The method works in ambient pressure and temperature conditions. On the other hand, the method has its limitations, for example, it depends on lot of sensitive factors such as the solubility of ligands, selection of solvents, and concentration of reactants. The method is also very time consuming and often requires the use of modulators like CTAB and PEG to fasten the process and to control the crystal growth rate. Another disadvantage of the method is its difficulty to be applied on an industrial scale where rapid bulk production is required [2,32]. In Table 2, it can be observed that the vapor diffusion technique is often modified by the usage of ultrasonication or by seed mediation.

Ultrasonication is another technique which has been widely mentioned for CD-MOF synthesis in food applications. The method is able to mitigate the challenges faced in using the vapor diffusion technique as it is fast, environmentally friendly, energy-efficient, and can be performed at room temperature. The cavitation generated by ultrasound eases the binding of cyclodextrin molecules with metal ions and the crystals obtained are nano-sized. In ultrasound, green solvents can be used to replace toxic organic solvents. The disadvantage associated with this technique is its high cost [2].

In this section we have focused on the synthesis of MOFs using the three native CDs.

### 3.1. Synthesis of α-CD MOF

**Figure 3:**
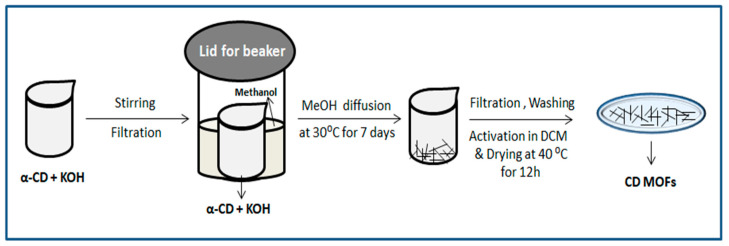
Schematic route of formation of MOFs by vapor diffusion method.

α-CD-MOF using sodium salt was synthesized by dissolving α-CD (1.46 g, 0.0015 moles) and NaOH (0.48 g, 0.012 moles) in deionized water (20 mL), and then MeOH (50 mL) was added to vapor diffuse into the solution for 7 days at 30 °C. The resulting crystals were filtered and washed three times with MeOH. To remove interstitial solvent, the α-CD-MOF crystals were immersed in dichloromethane for 3 days. The sample was dried in a 40 °C vacuum oven for 12 h. The method of preparing α-CD-MOF using potassium salt was the same, except that KOH (0.673 g, 0.012 moles) was used [33] (see Figure 3).

### 3.2. Synthesis of β-CD MOF

In the different literature, β-CD MOF has been mostly prepared with K^+^ ion due to its biocompatibility and nontoxic nature, which makes it easier to use in the food industry. Purified β-CD (1.1349 g, 0.99 mmol) and KOH (0.4488 g, 7.99 mmol) were dissolved in distilled water. The solution was then filtered and transferred to a beaker. The beaker was sealed in a tank containing 100 mL of methanol and kept for one week. In this time period, vapors from the methanol diffused into the solution and formed K-β-CD MOF [34]. A more defined method is stated by Jiang W et al., where β-CD (0.1418 g, 0.125 mmol) and KOH (0.056 g, 1 mmol) dissolved in H_2_O (5 mL). The molar ratio in most of the studies is maintained at β-CD:KOH = 1:8. This solution is then filtered through an organic membrane having a pore size of 0.45 μm into a small beaker containing 0.5 mL MeOH. This small beaker was kept inside a big container with 60 mL of MeOH, followed by vapor diffusion into aqueous solution at 50 °C for 12 h. After 12 h, a colorless and transparent liquid was obtained to which 5 mL MeOH solution (including 60 mg of PEG 20,000) was added and kept in a closed container. The addition of high molecular weight PEG is performed in order to control the size of the MOF crystals. PEG acts as a modulator that helps in nucleation as well as in achieving rapid crystal synthesis by avoiding aggregation which results in a smaller crystal size and higher surface to area ratio [35,36]. This colorless liquid was incubated overnight leading to the formation of K-β-CD MOF crystals. The crystals were collected after separation, washed with 15 mL ethanol twice and then suspended in dichloromethane for 3 days for activation. Ultimately, the crystals were desiccated overnight at 50 °C under vacuum conditions [4].

### 3.3. Synthesis of γ-CD-MOF

Majorly, the synthesis of γ-CD-MOFs was also performed using the vapor diffusion technique due to its simple procedure and effective results. One of the first works mentioned in the literature was reported by Smaldone et al. γ-CD and KOH were taken in a 1:8 mmol ratio and dissolved in 20 mL water. The solution was filtered and MeOH was allowed to vapor diffuse into the solution over a period of one week. The results were colorless cubic crystals which were isolated, filtered, and washed with MeOH and then left to dry in the air [37].

**Figure 4:**
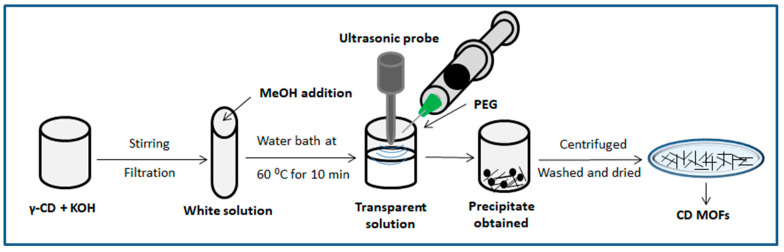
Schematic route of formation of MOFs by ultrasonication method.

Another method explained the use of ultrasonication for the production of γ-CD-MOF. In a work by Shen et al., γ-CD (648 mg, 0.5 mmol) and KOH (256 mg, 4.56 mmol) were dissolved in ultra-pure water (20 mL). The solution was filtered with a 0.45 μm filter membrane and stirred at room temperature. MeOH (12 mL) and the above solution were added in the tube to form a white solution. The tube was then transferred to a water bath at 60 °C for 10 min, and a clear and transparent solution was obtained. A probe ultrasonicator was used and the solutions were ultrasonically processed at a frequency of 20 kHz and a power of 540 W, a reaction under intermittent action for 10 min, the intermittent ultrasonic action mode was on for 2 s, off for 2 s, while PEG-8000 (256 mg) was quickly added after the start of the ultrasonic treatment to trigger the deposition of crystalline materials. In addition, the rapid synthesis and the regular morphology of the γ-CD-MOF crystal were inseparable from PEG-8000 as the excipient which was used as the size modulator for controlling the size and morphology of MOF crystals in aqueous systems. The crude product was obtained after the reaction. The crude product was allowed to stand for 1 h to obtain the white precipitate. The precipitate was centrifuged at 5000 rpm for 5 min and washed with MeOH 3 times, then the precipitate was dispersed in MeOH and dried at 50 °C under vacuum for 12 h [38] (see Figure 4).

**Table 2:** Common ways of synthesis of cyclodextrin-based metal–organic frameworks.

Metal Ion (Salt Used)	Ligand	Synthesis Technique	Conditions			Reference
			Mixing/ Ultrasonication Time	Temperature for Vapor Diffusion (°C)	Vapor Diffusion Time	
K^+^ (C_7_H_5_KO_2_)	α-CD	Vapor diffusion	6–8 h	R.T	3–7 days	[39]
K^+^ (KOH)	α-CD	Ultrasonication	30 min	After ultrasonication, solution was mixed with MeOH, heated at 60 °C, cooled to R.T, and PEG and MeOH were added to obtain crystals		[40]
K^+^ (KOH)	β-CD	Vapor diffusion	-	R.T	1 week	[41]
K^+^ (KOH)	β-CD	Vapor diffusion	-	50	12 h	[42]
K^+^ (KOH)	β-CD	Vapor diffusion	3 h	25	-	[43]
K^+^ (KOH)	β-CD	Vapor diffusion	3.5 h	R.T	3–5 weeks	[44]
K^+^ (KOH)	γ-CD	Ultrasonication	30 min	-	-	[40]
Rb^+^ (RbOH)	γ-CD	Vapor diffusion	-	R.T	1 week	[37]
K^+^ (KOH)	γ-CD	Vapor diffusion	6–12 h at 500 rpm	23	1 week	[45]
K^+^ (KOH)	γ-CD	Vapor diffusion	-	60	2 h	[46]
K^+^ (KOH)	γ-CD	Vapor diffusion	-	50	5 h	[47]
K^+^ (KOH)	γ-CD	Ultrasound assisted vapor diffusion	5 min	50	6 h	[48]
K^+^ (KOH)	γ-CD	Ultrasound assisted vapor diffusion	Different time (0, 5, 10, or 15 min)	50	6 h	[49]
K^+^ (KOH)	γ-CD	Seed-mediated methanol vapor diffusion	-	50	1 h	[50]
K^+^ (KOH)	γ-CD	Ultrasonication	30 min	After ultrasonication, solution was heated at 60 °C for 1 h and the PEG 20,000 was added to obtain crystals		[51]
K^+^ (KOH)	γ-CD	Vapor diffusion	6 h 500 rpm	R.T	3–7 days	[52]
K^+^ (C_7_H_5_KO_2_)	γ-CD	Vapor diffusion	6 h 500 rpm	R.T	3–7 days	[52]
K^+^ (KOH)	γ-CD	Ultrasonication	30 min	After ultrasonication, solution was mixed with MeOH, heated at 60 °C, cooled to R.T, and PEG and MeOH were added to obtain crystals		[40]
K^+^ (KOH)	γ-CD	Vapor diffusion	-	50	24 h	[53]
K^+^ (KOH)	γ-CD	Ultrasonication	30 min	After ultrasonication, solution was mixed with MeOH, heated and cooled to R.T, and PEG and MeOH was added to obtain crystals		[54]
K^+^ (KOH)	γ-CD	Seed mediated methanol vapor diffusion	-	50	6	[55]
K^+^ (KOH)	γ-CD	Seed-mediated ultrasonication	Different time (0, 3, 5, 10, and 15 min)	After ultrasonication, solution was mixed with MeOH to obtain crystals		[56]

## 4. Structural Aspects of CD-MOFs

Followed by synthesis, this section covers the structural aspects of CD-MOFs. Although, for the production of CD-MOFs, the most common metal ion used is potassium, synthetically obtained CD-MOFs possess different structural identities depending on various factors like the type of metal ion and also the type of metal salt (same cation, different anion) used.

### 4.1. Effect of Metal Ion on Structural Changes

Single crystal X-ray diffraction studies of α-CD-MOFs using different hydroxide salts like KOH, CsOH, and RbOH provided astonishing results. It was determined that α-CD-MOF-Na crystallizes in an orthorhombic crystal system with space group P2_1_2_1_2_1_ [33] while α-CD-MOF-Rb was a tetragonal system with P4_3_2_1_2 space group [57]. The α-CD-MOF-K was monoclinic with space group C 1 2 1 [33]. The different metal ion resulted in a different type of coordination between the metal ion and the corresponding oxygen atoms present in α-CD.

**Figure 5:**
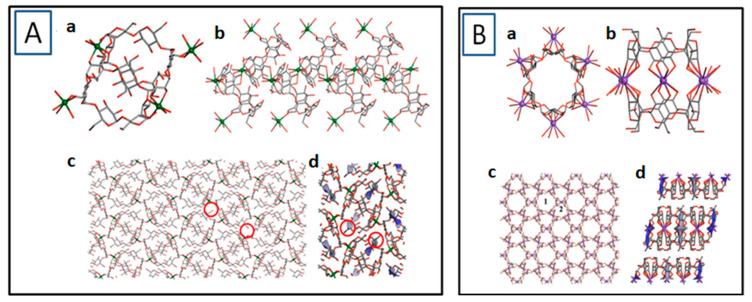
(**A**) Structure of (a) Na_2_(α-CD)_2_ subunit, (b) 1D chain produced by Na_2_(α-CD)_2_ subunit, (c) 3D structure of α-CD-MOF-Na, and (d) positioning of voids in α-CD-MOF-Na. Reprinted with permission [33]. Copyright 2020, American Chemical Society. (**B**) Structure of (a,b) K_6_(α-CD)_2_ subunit, (c) α-CD-MOF-K (1 represent the bigger the cavity with high volume and adsorption capacity and 2 represent smaller cavity), and (d) cavity in α-CD MOF-K. Reprinted with permission [33]. Copyright 2020, American Chemical Society.

The repeating unit of α-CD-MOF-Na consisted of one α-CD molecule, one Na^+^, and one water molecule. Two adjacent α-CD molecules were linked by Na^+^ ions to form an “8”-shaped Na_2_(α-CD)_2_ subunit [Figure 5A (a)]. The subunits were connected by the Na^+^ ion in order to produce 1D chains [Figure 5A (b)] which further connected the Na^+^ ion to produce 2D and 3D structures. As compared to the native α-CD molecule which possesses only one natural cavity, the MOF structure was found to be more porous as it not only contained the natural cavity of α-CD but there was formation of an extra cavity between two α-CD molecules. In comparison, α-CD-MOF-K consisted of two α-CD molecules and six K^+^, forming the K_6_(α-CD)_2_ subunit [Figure 5B (a,b)]. The K_6_(α-CD)_2_ subunits were connected to each other by K^+^ ions in the plane of the a and b-axes to form a layered structure. α-CD-MOF-K also formed two cavities but technically one of them was observed to be very small for adsorption and was not considered. Still, the higher cavity volume was observed in α-CD-MOF-K because the linkage of α-CD molecules by the K^+^ ion resulted in long channels which transformed their individual cavities into long tubular cavities. The surface area of α-CD-MOF-K was estimated to be 242.74 m^2^/g, much greater compared to 99.86 m^2^/g obtained in the case of α-CD-MOF-Na. Also, the void fraction in the case of α-CD MOF-Na was about 14.74%, which was less compared to 20% in α-CD MOF-K [33]. Ultimately, in the case of α-CD-MOF-Rb, two major motifs were observed, one in which the α-CD rings interacted with Rb^+^ ions through four α-1,4-linked D-glucopyranosyl residues while the other was coordinated to Rb^+^ ions by five of its six α-1,4-D-glucopyranosyl residues. Three secondary building units containing 4, 8, and 10 coordinated oxygen atoms with Rb^+^ ion were also observed. The BET surface area of α-CD-MOF-Rb was observed to be 820 m^2^/g [57].

**Figure 6:**
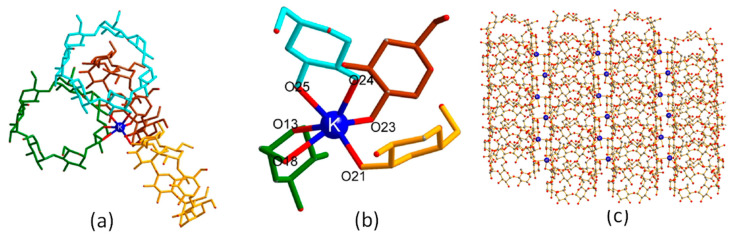
(**a**) The coordination situation of K-β-CD-MOFs (K^+^ and β-CD), (**b**) specific site coordination of K-β-CD-MOFs, (**c**) 3D structure of K-β-CD-MOFs. Reprinted with permission [4]. Copyright 2021, Elsevier.

K-β-CD MOFs structures developed using KOH as a metal salt were analyzed by Jiang et al. [4] and it was reported that K-β-CD MOFs crystallizes into a monoclinic crystal system occupying space group P_21_. The observed attachment of one K^+^ ion and six oxygen atoms was also reported by Charpin et al. [58]. It was seen that each independent β-CD molecule was connected to four K^+^ ions via one primary hydroxyl and two secondary hydroxyls, ring O-atom and the 6-OH groups (see Figure 6). The K–O bonds distance were in the range of 2.670–2.970. Similar observations were made for Cs-β-CD MOFs which also crystallized into a monoclinic crystal system occupying the P_21_ space group, although in this case, one Cs^+^ ion and seven oxygen atoms were linked through 2-,3-OH, the ring O atom, and 6-OH groups. Another example to be considered is Na-β-CD MOFs [59]. Single-crystal X-ray analysis reveals that the asymmetric unit of the Na-β-CD MOF is composed of two β-CD molecules, four Na cations, four OH^−^ ion, and one lattice waters. The Na^+^ ions possess different coordination modes and are referred to as Na1 and Na2. The formation of the 3D MOF structure starts with attachment of the 2,3-OH group from a- and d-glucopyranosyl of a β-CD molecule with 6-OH and 2,3-OH groups from e- and c-glucopyranosyl of an adjacent β-CD molecule by Na1, resulting in the formation of 1D chains. The adjacent 1D chains are fused together via Na2–O12 obtaining 1D double chains with double channel and left-handed helical structure. Finally, each left-handed helical chain is surrounded by four of the same channels via Na1–O10 (primary face 6-OH), forming the 3D CD-MOFs.

**Figure 7:**
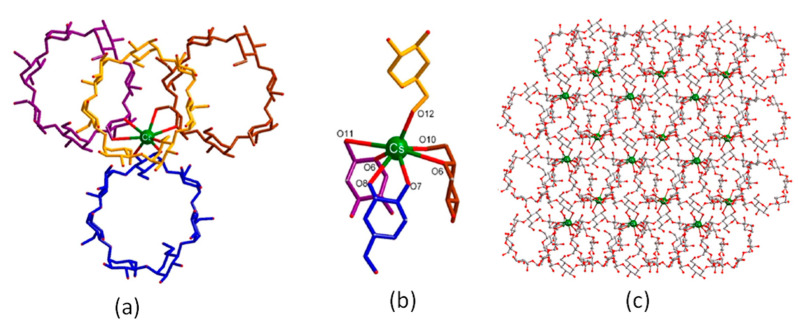
(**a**) The coordination situation of Cs-β-CD-MOFs (Cs^+^ and β-CD), (**b**) specific site coordination of Cs-β-CD-MOFs, (**c**) 3D structure of Cs-β-CD-MOFs. Reprinted with permission [4]. Copyright 2021, Elsevier.

**Figure 8:**
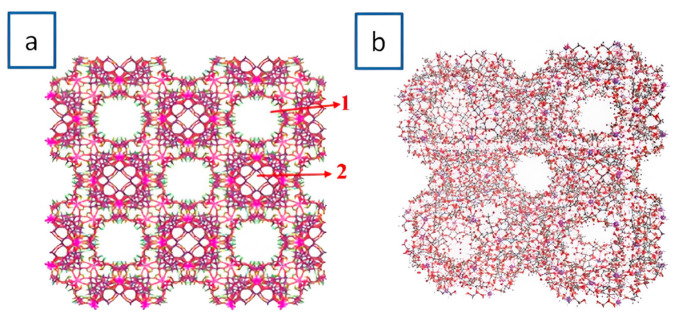
(**a**) Different types of cavities in K-γ-CD-MOFs, (**b**) 3d structure of K-γ-CD-MOFs. Reprinted with permission [49]. Copyright 2022, Elsevier.

For K-γ-CD-MOFs prepared by using KOH metal salt, the MOF crystallizes in the cubic space group P42_1_2 and has a repeating unit composed of two K^+^ ions, two water molecules, and one γ-CD molecule. The asymmetric unit in the unit cell consists of two γ-CD molecules and eight K^+^ ions, three of which the ion is coordinated by four oxygen atoms from two hydroxyl groups (−OH) in γ-CD, which connect two γ-CD molecules to form a sandwich structure: [K_8_(γ-CD)_2_] (Figure 7a–c). The [K_8_(γ-CD)_2_] subunits are connected by K^+^ ions and extend along the a- and b-axes to form a layered structure (Figure 8b). Thus, two different pores are formed, a larger cavity (marked 1 in Figure 8a) and a smaller cavity, between γ-CD molecules (marked 2 in Figure 8a).

### 4.2. Effect of Different Metal Salts (Same Cation but Different Anion)

In a work performed by Pan et al. [60], the effect of different potassium salts on the γ-CD-MOF was studied. KOH, KCl, and KAc metal salts were used for the formation of K-γ-CD-MOFs. The effect of different anions was very well explained; three γ-CD-MOFs crystal particles were observed to have different morphologies. The γ-CD-MOF prepared by KOH showed smoother and more regular particle surfaces, whereas the γ-CD-MOF synthesized with KCl or KAc had a higher degree of aggregation among the particles. From a microscopic point of view, the three γ-CD-MOFs varied in the space groups, and the cavity size and density were significantly different. The size distribution of the KOH-γ-CD-MOF and KCl-γ-CD-MOF cavities was 0.3–1.0 nm and 0.2–2.5 nm, respectively, and the number of cavities was relatively small. Compared with the KOH- and KCl γ-CD-MOFs, the KAc-γ-CD-MOF has more cavities with a size distribution of 0.2–1.0 nm and is dominated by small-size pores. In another work it was also reported that the KOH-γ-CD-MOF has a BET and Langmuir surface area of 1229 ± 76 and 1376 ±18, respectively, which was much higher than potassium benzoate-based KBz-γ-CD-MOF having a value of 417 ± 7110 and 607 ± 102. The reported average pore radius in the case of KOH-γ-CD-MOF (0.79 ± 0.01) was higher than that of KBz-γ-CD-MOF (0.40 ± 0.40). KBz-γ-CD-MOF was found to have almost doubled in size when compared with KOH-γ-CD-MOF, the reason being the lower nucleation rate due to the presence of benzoate anions in the solutions. The presence of the benzoate ion was also found in the cavities of the KBz-γ-CD-MOF, which resulted in a lower pore volume of 0.18 cm^3^/g compared to 0.50 cm^3^/g in KOH-γ-CD-MOF [52] (see Table 3).

## 5. Mechanism and Kinetics of Encapsulation/Loading and Release (Migration of Active Substance)

**Figure 9:**
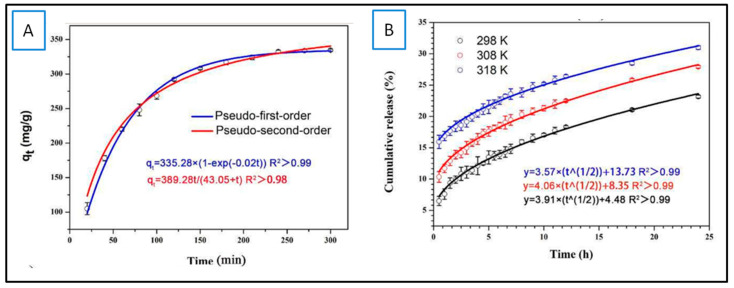
(**A**) Effect of contact time on load of EGCG, described through various models: pseudo-fist-order and pseudo-second-order. (**B**) Release profile of EGCG out of γ-CD-MOF-EGCGs, expressed by Higuchi model. Reprinted with permission [46]. Copyright 2023, Elsevier.

After the synthesis and structural understanding of CD-MOF, the next step usually involves the encapsulation of active principle inside the CD-MOF cavity for desired food application. Thus, it is important to evaluate the encapsulation and release kinetics of the active principle. Also, the understanding of the mechanism of the inclusion complex formation is crucial. Not a lot of work has been carried out on the kinetics of encapsulation/loading of active principles in CD-MOFs regarding the food applications, but a study by Wang et al. focused on the mechanism of loading epigallocatechin-3-gallate (ECGC) in γ-CD-MOFs, and it was understood through the simulation that the hydrogen bond, hydrophobic interaction, and π-stacking were main modes existing extensively to recruit EGCG into the frameworks. The adsorption of ECGC in γ-CD-MOF cavities was a two-step process involving rapid adsorption of almost 2 h at the outset, followed by a slow adsorption within 4 h, implying that the adsorption is a diffusion-driven process. To further define the release process, Lagergren’s pseudo-first-order and pseudo-second-order models were applied and it was revealed that adsorption data could be described better with the pseudo-first order model with a higher coefficient-of-determination (R^2^ > 0.99). Ultimately, it was speculated that the process of loading EGCG within γ-CD-MOFs inclines towards physisorption [46] (see Figure 9).

In a different work, it was observed that the amount of Glycyrrhizic acid (GA) encapsulated in γ-CD-MOF increased with the increase in GA concentration but reached a constant value after saturation of the cavities. After the application of temperature to this saturated GA-γ-CD-MOF, the encapsulation amount of GA in γ-CD-MOF started to increase again. For instance, at the highest GA level used, the amount of GA bound to the γ-CD-MOF was around 287 and 1027 μg/mg at 30 °C and 60 °C, respectively. This increase in encapsulation may have been partly because of the increase in water solubility of the GA with temperature, as well as an increase in the strength of any hydrophobic interactions. Ultimately, when temperature is further increased, the binding becomes weaker due to the increase in the entropy. The Langmuir isotherm model was applied to evaluate the maximum loading capacity; the model was suitable to assess the GA-γ-CD-MOF system with a high correlation coefficient (R^2^ > 0.98). The maximum loading capacity was found to be very high (Q_m_ = 2594 μg/mg) at a binding temperature of 60 °C [56].

**Figure 10:**
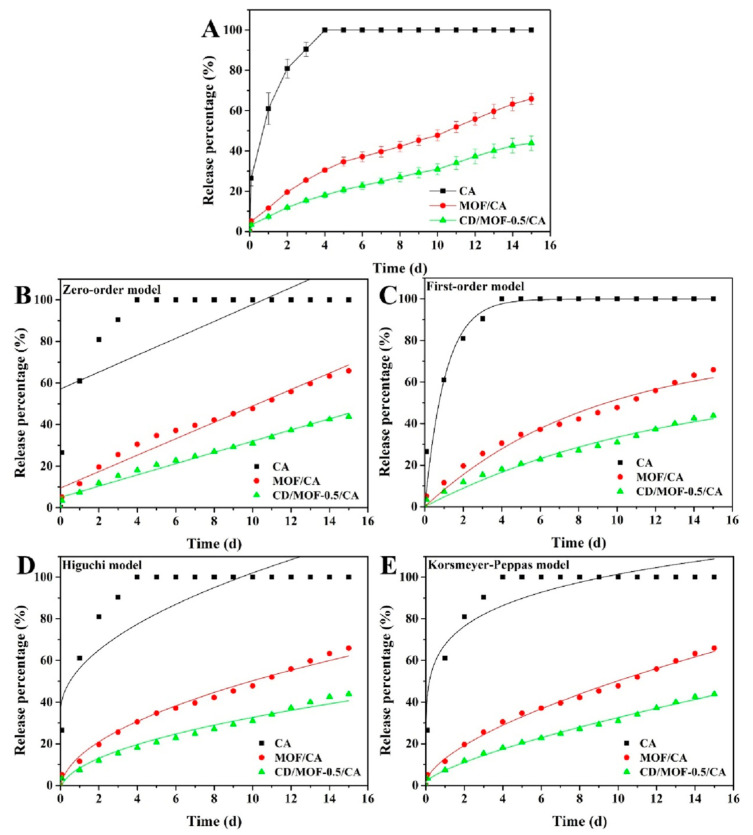
The release profiles of free cinnamaldehyde (CA), CA-γ-CD-MOFs, and CA-γ-CD-MOF-0.5CD at 8 °C: (**A**) zero-order (**B**), first-order (**C**), Higuchi (**D**), and Korsmeyer–Peppas model (**E**). Reprinted with permission [48]. Copyright 2022, Elsevier.

Different works have especially focused on the release kinetics of the active principle (encapsulated in CD-MOFs) on various food products in order to determine the effect of release rate on the preservation effect of the CD-MOF inclusion complex. In one study, caffeic acid-loaded γ-CD-MOFs (CA-γ-CD-MOFs) were used to study the release kinetics of caffeic acid in water, phosphate-buffered saline (PBS), and ethanol. It was observed that in water and PBS, CA-γ-CD-MOFs exhibited burst release while in ethanol a sustained release effect was observed. Further, the correlation coefficient R^2^ was evaluated using different models and it was found out that the order of the best fitting model was the Higuchi model > zero-order model > first-order model, and the possible underlying release mechanism of CA-CD-MOF in ethanol was the diffusion process [38]. In a similar way, release profiles of cinnamaldehyde (CA), CA-γ-CD-MOFs, and CA-γ-CD-MOF-0.5CD (where CD represents the volume of carbon dots added in ml) were evaluated and it was reported that the release rate order was CA > CA-γ-CD-MOFs > CA-γ-CD-MOF-0.5CD. To further understand the release mechanism, the correlation coefficient R^2^ was calculated and it was determined that at 8 °C, CA was best suited for the first-order model with the highest R^2^ value (0.9699). The release profile of CA-γ-CD-MOFs and CA-γ-CD-MOF-0.5CD were all well fitted by the Korsmeyer–Peppas model (R > 0.9945), indicating that the sustained release pattern was mainly controlled by diffusion. The “*n*” value in the Korsmeyer–Peppas equation depicts the diffusion mechanism. The “*n*” value of both CA-γ-CD-MOFs and CA-γ-CD-MOF-0.5CD was greater than 0.45 but less than 0.89, indicating that the release behavior was controlled by non-Fickian diffusion [48] (see Figure 10). 

A different study also mentioned about how the presence of water vapor in the environment triggers the disintegration of the topological structure γ-CD-MOF into γ-CD units and K^+^, and thus, carvacrol (CAR) molecules escape from the pores of γ-CD-MOFs. It was seen that at 98%RH, the release of CAR from CAR-γ-CD-MOFs/CS-CEL (Chitosan–Cellulose) film was kept at a higher rate, and it reached 99.71 ± 0.22% on the tenth day. While under 22% RH, the release pattern of CAR plateaued and there were only 14.71 ± 4.46% of CAR released on the twelfth day. On the twelfth day, the release of CAR reached 32.92 ± 3.06% and 85.43 ± 0.84% at 43% RH and 75% RH, respectively. To understand the release pattern and mechanism, four mathematical models are applied, namely, the zero-order model, first-order model, Higuchi model, and Ritger–Peppas model. The value of fitting correlation coefficient “R^2^” was calculated according to four models and the Ritger–Peppas model was found to be the most suitable model. Furthermore, the “*n*” value was lower than 0.45 at 20% RH, suggesting that the release of CAR followed the Fick diffusion, which was attributed to the CAR molecule attaching to the surface of the composite film. At the other tested humidities, “0.45 < *n* < 0.89” indicates that the release of CAR from CAR-γ-CD-MOFs/CS-CEL film complied with the non-Fick diffusion, which was caused by the synergistic effect of Fick diffusion and skeleton dissolution [61].

In summary, the release of active principles mostly followed the diffusion mechanism and the Ritger–Peppas model was most suitable to define the release profiles. Additionally, temperature and humidity play a huge role in the release of active molecules from the CD-MOFs: the increase in temperature and humidity was responsible for the increased release rate.

### 5.1. Effect of Different Metal Ions on Release Rate of Active Principles

**Figure 11:**
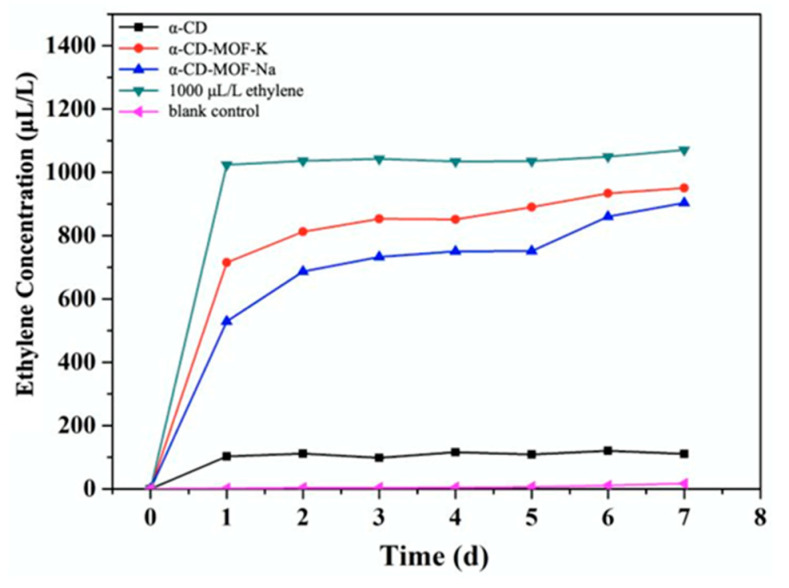
Ethylene concentration changes with respect to time. Reprinted with permission [33]. Copyright 2022, American Chemical Society.

The selection of metal ions also affects the release rate of active principles from CD-MOF. A comparison can be made on the basis of a study describing the release rate of ethylene molecules from α-CD-MOF-Na and α-CD-MOF-K. It was observed that the difference in the metal cation does not affect the release profile a lot (Figure 11). However, the concentration of ethylene molecules in α-CD-MOF-K was higher than α-CD-MOF-Na due to the presence of a larger cavity size [33].

**Figure 12:**
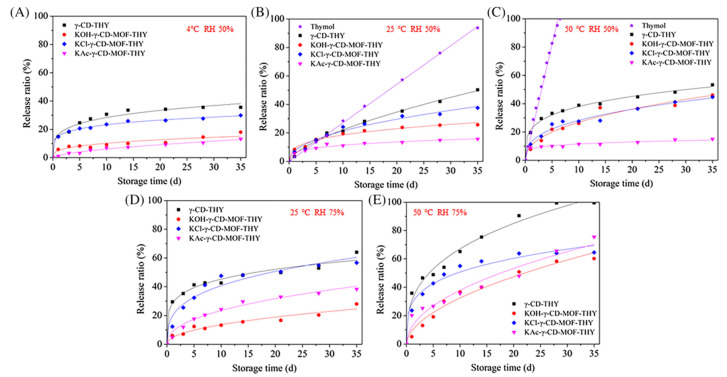
In vitro kinetics of thymol released from different γ-CD-MOFs at different temperatures and RH values stored for 0 to 35 days: (**A**) 4 °C, RH 50%, (**B**) 25 °C, RH 50%, (**C**) 50 °C, RH 50%, (**D**) 25 °C, RH 75%, and (**E**) 50 °C, RH 75%. Reprinted with permission [60]. Copyright 2022, Wiley.

In a similar study, two metal cations K^+^ and Cs^+^ were used for the formation of β-CD-MOFs and myricetin (MYR) was incorporated in it. It was detected that the maximum adsorption of MYR in β-CD-MOF-Cs (282.39 mg/g) was higher than β-CD-MOF-K (308.65 mg/g) due to a bigger cavity size. Also, the release profile in both cases was different. Since β-CD-MOF-K had a smaller cavity, the release of MYR initially was much slower and then later it followed a constantly increasing rate, while β-CD-MOF-Cs, due to its bigger cavity size, started releasing MYR instantly and later followed a similar profile as β-CD-MOF-K [4]. The effect of anions on the release profile is also evaluated in a study by Pan et al. [60] and it was noticed that thymol (THY) incorporated in γ-CD-MOF showed a slower release rate than THY-γ-CD and free THY. The release rate under the influence of temperature and relative humidity are shown in Figure 12

### 5.2. Effect of CD-MOFs on the Stability of Possible Active Principles

**Figure 13:**
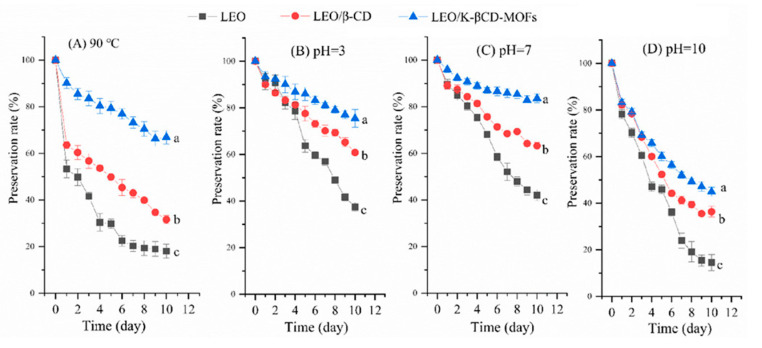
Thermal (**A**) and pH stabilities (**B**–**D**) of lavender essential oil (LEO), LEO/β-cyclodextrins (LEO/β-CD), and LEO/metal–organic frameworks based on β-cyclodextrin and potassium cation (LEO/K-βCD-MOFs) inclusion complexes [34].

In the previous section, we talked about the mechanism and kinetics of encapsulation and release. The main goal of CD-MOF production is to stabilize the volatile active principle and deliver it. Stability of active principles in CD-MOFs under different conditions are important to be studied. The thermal and the pH stabilities of lavender essential oil (LEO) were evaluated and it was reported that the CEO-β-CD-MOF complex showed the highest thermal stability. After 24 h heat treatment, the preservation rate of LEO decreased to 53.27%, while the rate of LEO in the LEO/K-βCD-MOF complex still remained at 90.13%. For 10 days the same trend was followed and it was confirmed that the thermal stability of LEO significantly improved after encapsulation in K-βCD-MOF. The influence of pH on stabilities of LEO, LEO/βCD, and LEO-K-βCD-MOF was evaluated and it was found out that the stability of LEO in LEO/βCD and LEO-K-βCD-MOF was much stronger in acidic and neutral environments compared to alkali environments [34] (see Figure 13).

The influence of light, temperature, and oxygen on the stability of catechin (CA) in CD-MOFs was also studied, and observations pointed out that βCD-MOFs increased the stability of catechin after encapsulation. CA, which is usually degraded when exposed to UV/Visible radiation, showed lower antioxidant reduction after encapsulation in CD-MOF. The antioxidant retention (%) of CA was 45.5% after 30 days of exposure, while for the CA/α-CD-MOFs, CA/β-CD-MOFs, and CA/γ-CD-MOFs it was 75.8%, 76.85%, and 79.98%, respectively. The thermal stability of CA also improved after encapsulation in CD-MOF; results indicated that encapsulation of CA into CD-MOFs provided protection against temperature up to 120 °C. The stability of free CA which oxidizes easily was also evaluated after exposure to oxygen. The antioxidant retention (%) of CA was only 19.8% after exposure to air for 30 days while the antioxidant retention (%) of the CA/CD-MOFs still remained above 80%, indicating that CA was effectively protected against oxygen by CD-MOF encapsulation [40].

So far, we can conclude that the CD-MOFs are affected by the increase in relative humidity; from this we can infer that CD-MOFs are surely water soluble and their use in applications where the presence of water is high is a waste. However, in most food applications, especially related to packaging where the humidity is controllable, the usage of CD-MOFs provides the huge advantage of delivering active molecules which are mostly non-soluble in nature. One study demonstrates that the encapsulation of polyphenols in β-CD-MOFs improved its solubility in various solvents, particularly in water (Table 4). The spontaneous dispersibility of the curcumin (Cur) was also tested after incorporation in Nano-γ-CD-MOFs. During the process of dissolution, Cur and Cur-γ-CD sank fast and then deposited in the bottom of the bottle when added into deionized water while in the case of Cur-γ-CD-MOF, a clarified solution, was obatined [51].

## 6. CD MOFs Applications in Food Industry

The direct usage of highly volatile compounds like essential oils is difficult in any application. There is a need to provide them with the stability required during the process of product manufacturing without the loss of their inherent properties. Encapsulation is an easier and highly effective process while dealing with compounds like essential oils. The active molecule (guest molecule) can be entrapped inside the cavities of CD-MOFs overcoming the issues that exist in the direct usage of active molecule. Some of the improvements that are observed are as follows [62].

- The diffusion and volatility (in the case of volatile substances) of the included guest can decrease strongly.
- The complexed substances, even gaseous substances, can be entrapped in a carbohydrate matrix forming a microcrystalline or amorphous powder.
- The complexed substances can be effectively protected against heat decomposition, oxidation, and any other type of reaction, except against those with the hydroxyl groups of cyclodextrin, or reactions catalyzed by them.

The above-mentioned positive changes give the liberty of using different active principles for various applications in the food-based industry; some of them are mentioned in Table 5. However, it is also worth noticing that for the MOFs to be a useful system, they require different matrices to be incorporated and to be used in different applications.

In various studies, it was observed that CD-MOFs have been basically used in food applications either directly or by incorporating them in films or using them in sachets and pouches. In a study, polylactic acid (PLA)-based small bags were used to contain geraniol-incorporated β-CD-MOF (Gr-β-CD-MOF) and were kept in a Polyethylene container along with the emperor bananas. The preservative effect of the Gr-β-CD-MOF was analyzed and it was determined that the system could be used in potential food storage-based applications [63]. In a similar fashion, non-woven bags were filled with γ-CD MOFs containing cinnamaldehyde (CA). These CA-γ-CD MOFs were then transferred into UV-sterilized fresh-keeping polypropylene boxes along with the fresh cut cantaloupes and ultimately it was established that CA-γ-CD MOF was able to inhibit the growth of *E. coli* on cantaloupes [48]. In a different study, hexanal-incorporated β-CD-MOF were converted into pellets and were affixed to a box containing mangoes. The weight loss of the mangoes decreased and the shelf life improved [64]. Anchoring of γ-CD MOFs was also performs on Chitosan–Cellulose (CS-CEL) films to form γ-CD MOF/CS-CEL composites. These composites were further incorporated with carvacrol. The films were then used for the preservation of strawberries and proved to be effective [61]. Composite films of γ-CD-MOFs encapsulating curcumin (CUR) and Pullulan and trehalose (PUL/TRE) were also developed. For the film formation, a casting method was used. CUR-γ-CD-MOFs were added in Pullulan and trehalose (PUL/TRE) solution, followed by stirring at 50 °C for 30 min and ultimately casting of the film. The films were then used for the preservation of Centennial Seedless grapes [47]. Coating of 2% sodium alginate and anthocyanin containing γ-CD-MOFs were also applied to fresh grapes and were compared with the control. It was reported that the coated grapes had lower weight loss and better shelf life [53].

**Figure 14:**
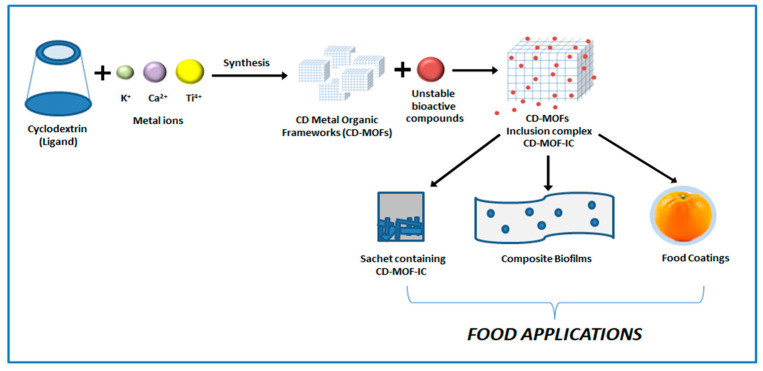
A schematic diagram of CD-MOF synthesis and its potential food industry applications.

Further effects of the above-mentioned works on food applications are mentioned in Table 5 and the Characterization section. Figure 14 below show a schematic depicting formation of inclusion complex and various ways through which it can be used in food applications.

**Table 5:** Overview of existing and potential application of CD-MOFs in food industry.

MOF	Active Compound	Application	Important Observations	Reference
α-CD MOF	Ethylene gas	Accelerated fruit ripening	MOF-ethylene complexes had controlled ethylene-release for accelerated fruit ripening.	[33]
α-CD MOF	Catechin	Potential application in food packaging	CD-MOFs protected catechin against light, oxygen, and temperature, thus improving its storage stability. Catechin encapsulated within CD-MOFs exhibited superior bioavailability.	[40]
β-CD MOF	-	Herbicide adsorption and potassium replenishment	The maximum adsorption capacities of four herbicides were in the range of 261.21–343.42 mg/g^−1^. The herbicide removal percentage was in the order: MET > PRE > ALA > ACE.	[65]
β-CD MOF	Hexanal	Preservation of mangoes	Treated fruit remains fresh until 2 weeks after storage. They possessed higher firmness and had lower weight loss.	[64]
β-CD MOF	Catechin	Zein-based packaging film	Zein films with catechin-loaded β-CD MOFs possessed better physical properties, antibacterial characteristics, and a more steady release profile for catechin compared to normal Zein film containing catechin.	[66]
β-CD MOF	Clove essential oil (CEO)	Preservation of Chinese bacon	Decrease in the lipid oxidation of bacon due to the increasing inhibitory effect of CEO after encapsulation in β-CD-MOF. Apart from that, the free radical scavenging activities and thermal and pH stabilities were also better in the case of CEO/β-CD-MOFs than just CEO.	[67]
β-CD MOF	Lavender essential oil (LEO)	Potential application in food packaging	LEO/K-βCD-MOFs were proved to be more thermally and acid-base stable than LEO, and its intracellular antioxidant effect was also significantly improved by encapsulation.	[34]
β-CD MOF	Thymol (THY)	Preservation of cherry tomatoes	The decay index of whole cherry tomatoes treated with γ-CD-MOF-THY decreased from 67.5% (control group) to less than 20% during storage at room temperature for 15 days.	[43]
β-CD MOF	Polyphenols	Potential application in food packaging	The stabilities and solubility’s of ALP were significantly improved compared to when encapsulated in β-CD-MOFs compared to β-CD, suggesting the potential of β-CD-MOFs as better carriers than β-CD for polyphenols in food industry applications.	[41]
β-CD MOF	Origanum Compactum essential oil (OCEO)	Potential application in food packaging	Compared to βCD, K-βCD-MOFs displayed higher encapsulation efficiency. Antioxidant capacity of OCEO was significantly enhanced in the presence of K-βCD-MOFs.	[68]
β-CD MOF	-	Extraction of Organochlorine pesticides from honey samples	CD-MOF/TiO_2_ has good selective enrichment ability for OCP and is suitable for the D-SPE pre-treat of honey sample analysis.	[69]
γ-CD MOF	Anthocyanins	Grape preservation	Grapes coated with sodium alginate + CD-MOFs containing anthocyanin showed gradual decrease in weight loss after 10 days. The firmness and epidermal puncture value of the grapes was also high with this coating. Brix value was found to be less compared to others.	[53]
γ-CD MOF	Ethylene gas	Accelerated ripening as well as preservation of bananas	Polycaprolactone nanofibers containing γ-CD-MOF and TiO_2_ were used. The γ-CD-MOF were encapsulated with ethylene and helped in the accelerated ripening of bananas while TiO_2_ under the action of UV helped to degrade ethylene, prolonging the shelf life of the bananas.	[70]
γ-CD MOF	Carvacol	Chitosan–Cellulose(CS-CEL) active packaging film	CS-CEL films containing Carvacol-γ-CD MOF showed the lowest weight loss in strawberries compared to other conditions. Also, Carvacol-γ-CD-MOFs/CS-CEL composite film showed the lowest firmness loss, highest TSS value, and lowest pH change.	[61]
γ-CD MOF	Cinnamaldehyde	Preservation of fresh cut cantaloupes	CD/MOF containing cinnamaldehyde (CA) and carbon dots improved the shelf life of the fresh cut cantaloupes and maintained the quality of the fruit. It was observed that CD/MOF-0.5 (amount of carbon dots)/CA exhibited a strong and long-lasting antibacterial activity when tested against *E. coli* in vitro and on fresh-cut cantaloupes.	[48]
γ-CD MOF	Curcumin	Preservation of Centennial Seedless grapes (CSg) through Pullulan and trehalose (Pul/Tre) composite film containing curcumin-γ-CD-MOF	The naturally placed CSg began to rot on the 4th day, while the CSg coated with Pul/Tre film rot on the 8th day with a shrunken surface and severe dehydration. However, the appearance of CSg coated with Cur-CD-MOFs-Pul/Tre film was still largely unaltered on day 10.	[47]
γ-CD MOF	-	Ethylene absorber for improving postharvest quality of kiwi fruit	The fruit in the γ-CDMOF-K group did not decay over the whole storage period, maintained a good appearance, and remained edible.	[49]
γ-CD MOF	Octadecenylsuccinic anhydride (ODSA)	Pickering emulsions coating and package paper for fruit preservation	The uncoated bananas experienced a 27.5% weight loss after 9 days, whereas the sample coated with a 10% ODSA emulsion had just 15.6% weight loss. Similarly, the weight loss also reduced in ODSA emulsions containing ODSA modified γ-CD-MOFs.	[71]
γ-CD MOF	β-carotene	Development of high internal phase emulsion (HIPE)	CD-MOF offers a safeguarding matrix for β-carotene, reducing the degradation and enabling a modulated release profile.	[72]
γ-CD MOF	Vitamin A palmitate	Encapsulation of vitamin A palmitate (VAP) for delivery as a food supplement	The half-life (t_1/2_) vitamin A in γ-CD-MOFs/VAP was recorded to be 20.5 days which is a 1.6 time increase compared to BASF vitamin A powder (t_1/2_ = 13.0 days) and a 2.6 time increase compared to physical mixture (t_1/2_ = 7.9 days), respectively.	[73]

## 7. Characterization of CD-MOFs

In this section, we have focused on some of the most useful techniques that are being used for the analysis of the MOF structure, especially for the characterization of the CD-MOF inclusion complex. These techniques help us to confirm the formation of inclusion complex by showing the presence of active principles like essential oils inside the MOF cavities. This section also informs about the evaluation of various properties that shows the effect of CD-MOF inclusion complex on the preservation of food products.

### 7.1. Thermal Analysis

a. Thermogravimetric Analysis (TGA)

**Figure 15:**
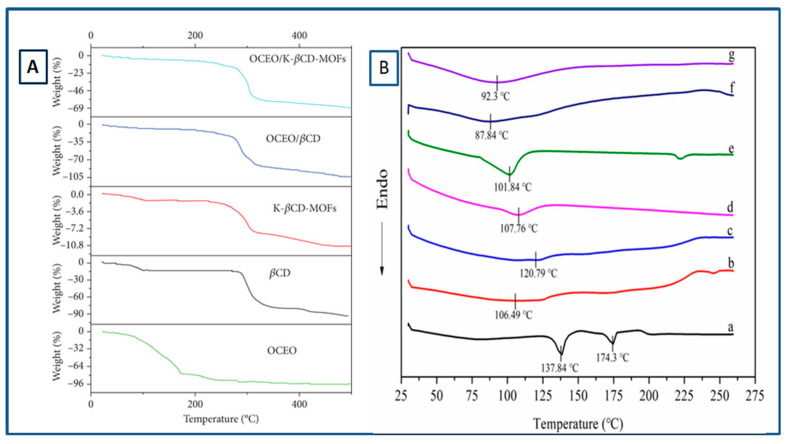
(**A**) TGA curve of raw OCEO and after its encapsulation in β-CD and β-CD-MOF [68], (**B**) DSC curves for (a) catechin(CA), (b) α-CD-MOFs, (c) CA/α-CD-MOFs, (d) β-CD-MOFs, (e) CA/β-CD-MOFs, and (f) γ-CD-MOFs and (g) CA/γ-CD-MOFs. Reprinted with permission [40]. Copyright 2022, Elsevier.

TGA is an important characterization technique to understand the thermostability of any type of sample. It helps us to identify the degradation temperature of the compound. We can also analyze the presence of various compounds inside a sample and also know the quantities of different compounds present. Changes in weight loss and shifts in degradation temperature or evaporation temperature indicate towards the formation of an inclusion complex. In a study by Hu et al., a lower degradation temperature for CD-MOFs was observed compared to native CDs which attributed to the weaker metal coordination bond and porous structure of the MOFs; the results indicated the formation of K-CD framework [44]. The thermal stability of CEO was found to be better when encapsulated in K-β-CD MOFs compared to just β-CD. After heating for 10 days, the rates of CEO/β-CDMOFs and CEO/β-CD remained at 63.35 and 50.23%, respectively; however, that of the free CEO was only 24.03% [67]. Similarly, in the case of LEO, a higher preservation rate was observed for LEO/K-βCD-MOFs (66.8%) compared to LEO/β-CD (31.50%) and native LEO (18.00%) after 10 days. In Figure 15A, it can be seen that encapsulation of Origanum compactum essential oil (OCEO) in β-CD-MOF improved the thermal stability of OCEO.

b. Differential Scanning Calorimetry (DSC)

Another technique reported to clarify the interaction between guest and host is differential scanning calorimetry (DSC). It is generally observed that the cyclodextrin cavity affects the thermal behavior of inclusion complexes, thus leading to a shift in their endothermic peaks compared to pure compounds. The shifting, broadening, and appearance of new peaks or disappearance of certain peaks in the host may be due to evaporation, oxidation, decomposition, melting, or polymorphic transition, suggesting complex formation. Inclusion complex formation generally causes the reduction or absence of endothermic peaks of the host (at the temperature of its boiling and melting point). A work confirmed the formation of inclusion complex CA-β-CD through the shift in the peak for different CD-MOFs. It can be seen in Figure 15B that the α-CD-MOFs, β-CD-MOFs, and γ-CD-MOFs displayed a wide endothermic peak at 106.49, 107.76, and 87.84 °C which were shifted to 120.79 °C for CA/α-CD-MOFs, 101.84 °C for CA/β-CD-MOFs, and 92.3 °C for CA/γ-CD-MOFs after CA loading. This change in the peak value can be reasoned with the encapsulation of CA in the cavities of porous MOF structures which altered the thermal properties [40]. Similarly, the formation of curcumin-γ-CD-MOFs was also evident from the disappearance of the melting peak of curcumin (174.35) when encapsulated in γ-CD-MOFs [51]. 

### 7.2. Microscopy

**Figure 16:**
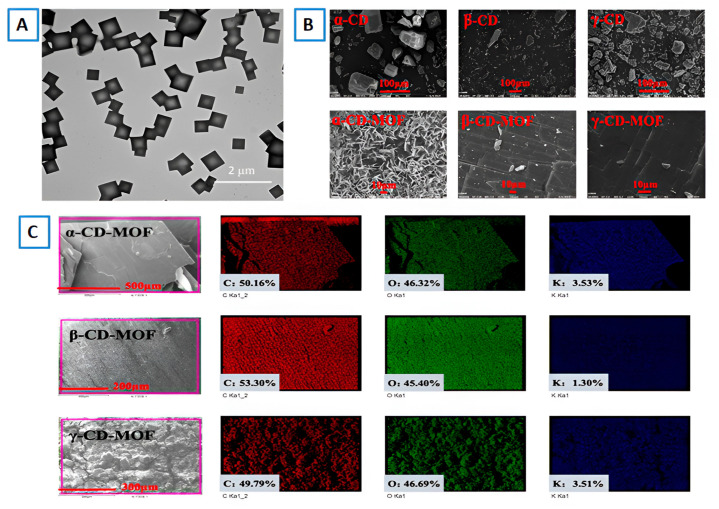
(**A**) TEM images of γ-CD-MOFs. Reprinted with permission [50]. Copyright 2019, American chemical society. (**B**) SEM images of that α-CD, β-CD, γ-CD, and their MOFs. Reprinted with permission [44]. Copyright 2021, Elsevier. (**C**) EDX data of α, β, and γ CD MOFs. Reprinted with permission [44]. Copyright 2021, Elsevier.

Microscopic techniques like scanning electron microscopy (SEM) and transmission electron microscopy (TEM) are the most initial techniques to use for MOF characterization. These techniques enable us to evaluate the particle size and shape of the formed framework. Both of the techniques can be further extended for better analysis of samples. For example, SEM can be used along with EDX for elemental mapping of the MOFs. The techniques are also able to provide an instant visual representation of change in the morphology of MOFs due to variations in different factors responsible for the final structure of MOFs. Shen et al. showed the effect of varying ultrasonic power, ultrasonication time, and incubation temperature on the final structure of γ-CD-MOF. It was observed that the change in incubation temperature resulted in the formation of MOFs with different sizes. When the temperature increased, the particle size of the samples also increased; this phenomenon may be attributed to the fact that the nucleation rate was inversely proportional to the temperature during the crystallization process [38]. In another work, it was detected that β-CD-MOF and γ-CD-MOF were highly crystalline and possessed cuboid and cubic shapes, respectively, while α-CD-MOF had flaky crystals of uniform size (Figure 16B). Elemental mapping of CD-MOFs (Figure 16C) also provided information about the homogenous distribution of C, O, and K [44]. TEM was also performed to confirm that γ-CD-MOFs displayed a uniform cubic shape (Figure 16A), and the mean particle size was approximately 190 nm [50].

### 7.3. X-Ray Diffraction

**Figure 17:**
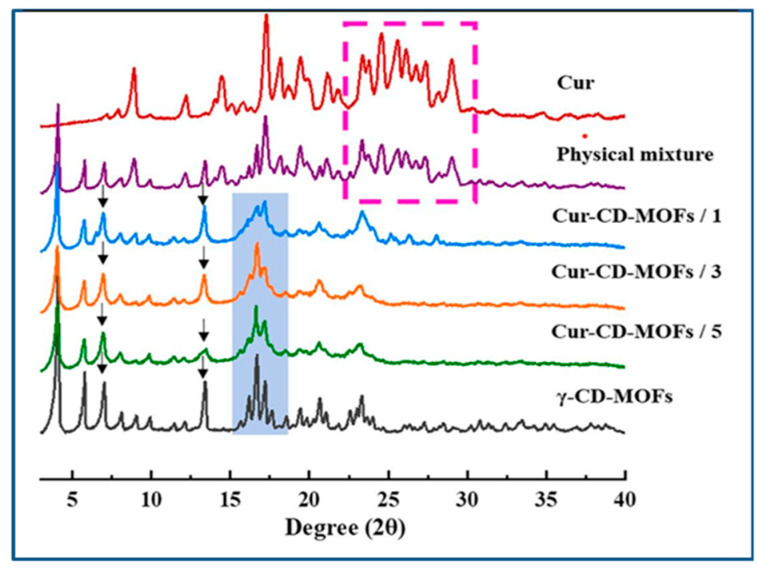
XRD pattern of curcumin and its inclusion complexes with γ-CD-MOFs. Reprinted with permission [54]. Copyright 2021, Elsevier.

X-ray diffraction is considered to be one of the best techniques for studying the structure of the metal–organic frameworks. The technique detects the crystallinity of the formed MOF. The sharpness of the peaks indicates the crystalline nature of the sample. The shifts in the peaks also help us to understand the formation of inclusion complexes with the MOF. Jiang et al. found that both K-β-CD-MOF MOFs and Cs-β-CD-MOFs possessed high crystallinity [74] with diffraction peaks at 4.6, 6.36, 9.24, 10.42, 12.2, 18.54 and 6.68, 9.0, 11.7, 13.2, 16.38, 18.8, respectively [4]. Their comparison of the experimental diffraction pattern with the simulated implied that the MOFs were formed. These data are consistent with a previous report which was also verified the XRD spectra of K-β-CD-MOF [42,75]. XRD analysis of γ-CD-MOFs and curcumin-loaded γ-CD-MOFs (Cur-CD-MOF) are shown in Figure 12. It can be observed that the characteristic diffraction peaks of curcumin were present when it was physically mixed with γ-CD-MOF but the peaks disappeared when compared with Cur-CD-MOF indicating the successful encapsulation of curcumin in the MOF cavities [54] (see Figure 17).

### 7.4. Spectroscopy

a. Fourier Transform Infrared (FT-IR)

**Figure 18:**
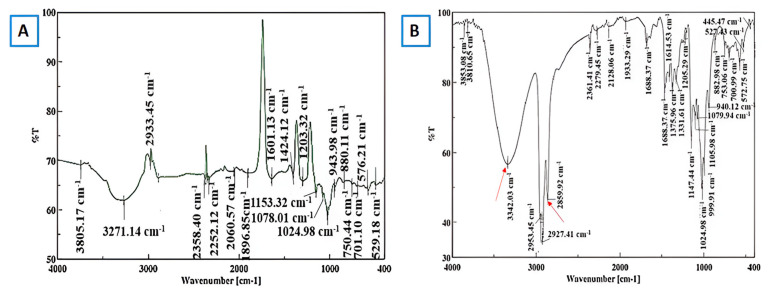
FT-IR spectra of MOF crystals (**A**) before hexanal encapsulation and (**B**) after hexanal encapsulation (red arrows represent the hexanal related peaks). Reprinted with permission [64]. Copyright 2021, American Chemical Society.

Fourier Transform Infrared (FTIR) spectroscopy is typically used to identify functional groups. FTIR is thus used initially during MOF synthesis to identify impurities and residual reactants on external surfaces and pores in the interior. Non-reacted organic linkers and coordinated solvents in the MOF structures can easily be identified through FTIR [76]. The formation of an intra-molecular hydrogen bond/complex formation between the cyclodextrin and host moieties can be confirmed with the aid of this technique [74]. Pan and his group reported the shifts in the wavelength when the native γ-CD was converted to γ-CD-MOF and also when the γ-CD-MOF was used to form an inclusion complex with thymol. Compared with those in γ-CD, the O–H stretching vibration peak (3750–3000 cm^−1^) and C–H stretching vibration peak (2900–3000 cm^−1^) in γ-CD-MOFs moved slightly to a low wavenumber, indicating that the –OCCO–coordination group of CDs formed a good topological structure with K^+^, and the hydrogen bond between molecules was strengthened. Compared with those of γ-CD-MOFs, the O–H and C–H stretching vibrations of the inclusion complex (IC) shifted slightly to the lower wavenumber. Compared with those of free thymol, the O–H and C–H stretching vibrations of IC shifted to a higher wavenumber. Alternatively, for ICs, the aromatic-ring characteristic peak (1600–1400 cm^−1^) of thymol and the characteristic peak of the 1,2,4-tri-benzene ring (900–650 cm^−1^) weakened, indicating that thymol was trapped in the hydrophobic cavity, which increased the hydrogen bond strength of γ-CD-MOFs during the composite process [60]. Results based on similar phenomena were established by Jiang et al. working with catechin (CAT) and β-cyclodextrin inclusion complexes. It was observed that after the inclusion of catechin in the β-CD-MOF, the O–H peak which was previously at 3382 cm^−1^ shifted to a lower wavelength of 3373 cm^−1^, indicating the formation of hydrogen bonding interactions between CAT and β-CD-MOFs. Moreover, the peaks at 1520 and 1466 cm^−1^, representing the alkene part of CAT, disappeared in CAT-β-CD-MOFs, indicating that CAT was completely trapped in the pores of β-CD-MOFs [66]. Figure 18 shows FT-IR results which prove the formation of inclusion complex after the encapsulation of hexanal in β-CD-MOFs.

b. Raman Spectroscopy

**Figure 19:**
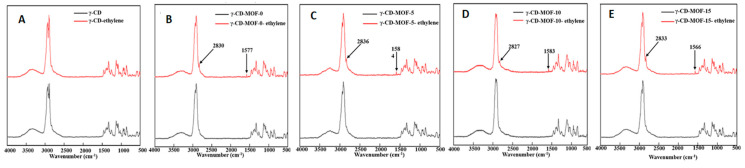
Comparison of Raman spectra of γ-CD-MOF before and after ethylene absorption (**A**) γ-CD shows no sign of ethylene absorption. (**B**–**E**) Absorption of ethylene in γ-CD-MOF prepared at different ultrasonic times. Reprinted with permission [49]. Copyright 2023, Elsevier.

Raman spectroscopy is a technique complementary to IR. Raman spectroscopy is an inelastic scattering technique, while IR spectroscopy is an absorption-based, and most often a mode that is IR active (where there is a change in the dipole moment) is Raman inactive (no change in polarizability) and vice versa. Hence, to obtain a complete understanding, it is best to employ both methods. Raman spectroscopy can help us to evaluate the structural changes in MOFs which involve the displacement of lightweight atoms such as hydrogen, carbon, and oxygen [77]. It can also provide evidence regarding the formation of inclusion complexes with MOFs and other porous compounds. Liu et al. used Raman spectroscopy and detected a great degree of disordered carbon in the calcined β-CD-MOF compared to β-CD-MOF, which indicated the loss of the crystalline structure due to carbonization of β-CD-MOF at a high temperature [65]. In another work, Raman spectra provided information about the successful encapsulation of the ethylene gas in the γ-CD-MOF which was impossible in γ-CD. Characteristic peaks were observed at 1570 cm^−1^ resulting from C=C bonds and 2826 cm^−1^ from the C–H bonds in ethylene-loaded γ-CD-MOF, which were previously absent in γ-CD-MOF [49] (see Figure 19).

c. Nuclear Magnetic Resonance (NMR)

**Figure 20:**
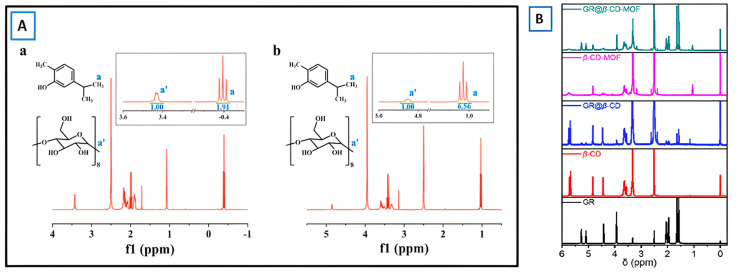
(**A**) ^1^H NMR of (**a**) CAR-γ-CD and (**b**) CAR-γ-CD-MOF. Reprinted with permission [61]. Copyright 2023, Elsevier. (**B**) The 1H NMR spectra of geraniol (GR), β-CD, GR-β-CD, β-CD-MOF, and GR-β-CD MOF. Reprinted with permission [63]. Copyright 2024, American Chemical Society.

Nuclear Magnetic Resonance (NMR) spectroscopy is a well-established tool for the investigation of various types of porous materials. It is an important characterization technique to detect the presence of any guest inside the MOF cavity (host) and also to understand the host–guest interactions. The contents of encapsulated carvacrol (CAR) in γ-CD and γ-CD-MOF were measured by ^1^H NMR spectra (Figure 20A) [61]. The encapsulated content of CAR was calculated by the peak area integral ratio of –OCH– of γ-CD unit and –CH_3_ of CAR. For example, the results showed that the integral ratio of the characteristic peak (a’) in γ-CD and γ-CD-MOF to CAR (a) was 8:1.91 and 8:6.56, respectively. Therefore, the molar ratio of γ-CD and γ-CD-MOF to CAR was approximately 1:0.64 and 1:2.19, respectively. The improved encapsulation capacity of γ-CD-MOF for CAR may be owing to its multiple pores. In addition, the polarity of γ-CD-MOF and CAR may also facilitate the strong interaction between them, thus promoting the encapsulation of CAR. In another work, it was also evaluated that compared to β-CD, the ^1^H chemical shifts in β-CD-MOF exhibited changes possibly due to the introduction of potassium ions coordinating with hydroxyl groups in β-CD, resulting in a decrease in their respective chemical shifts [63]. The ^1^H NMR spectra showed the presence of geraniol in GR-β-CD-MOF and GR-β-CD (Figure 20B). The insertion of guest molecules in β-CD-MOF and β-CD will change the chemical shift in H. The inclusion of geraniol with β-CD-MOF and β-CD induces a downfield shift in the chemical shifts, indicating successful synthesis of the GR-β-CD MOF and GR-β-CD complex.

### 7.5. UV–Visible

**Figure 21:**
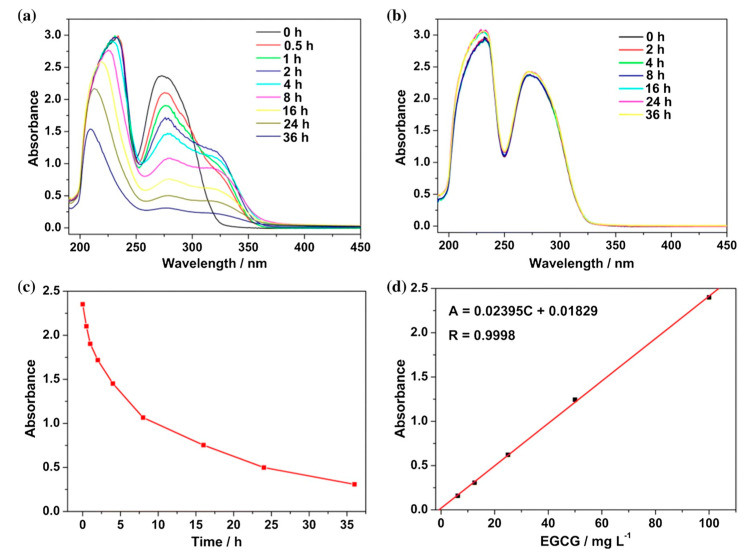
UV–Vis absorption spectra of EGCG in the presence (**a**) and absence (**b**) of CD-MOF in ethanol with time. (**c**) Variation in absorbance at 276 nm of EGCG in the presence of CD-MOF and (**d**) the calibration curve of EGCG in ethanol. Reprinted with permission [78]. Copyright 2019, Springer Nature.

While the former techniques focus mainly on the characterization of MOFs and provide necessary information about the formation of the inclusion complex (encapsulation of active principle inside the MOF), UV-Vis plays a crucial role in providing information about the release kinetics of the compound loaded in the MOF cavities. The rate at which the active molecules leave the MOF cavities is an important factor in applications where delivery has to be maintained for a longer time; UV-Vis helps clarify that delivery pattern for an active molecule. Apart from that, the adsorption efficiency and kinetics of systems like MOFs can also be studied and compared with other systems. It was found that the adsorption peak of (-)-epigallocatechin gallate (EGCG) continuously showed a drop for the time when samples of γ-CD-MOF were present in its solution (Figure 16). These hypochromic shifts indicated the inclusion of ECGC into the MOF cavities [78] (see Figure 21).

### 7.6. Antioxidant Activity

**Figure 22:**
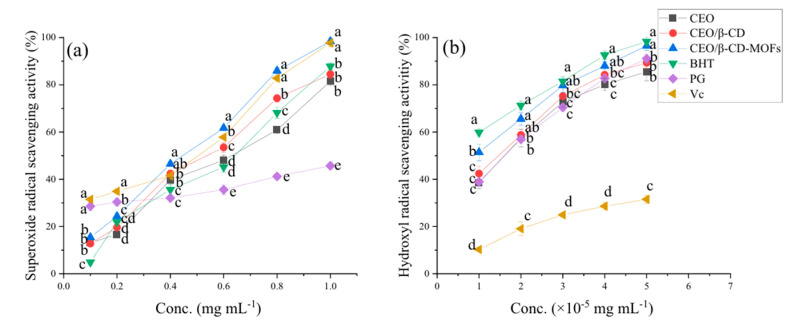
Antioxidant activities of CEO, CEO/β-CD, and CEO/β-CD-MOFs inclusion complexes and synthetic antioxidants. Superoxide anion scavenging activities (**a**) and hydroxyl radical scavenging activities (**b**) of CEO, CEO/β-CD, and CEO/β-CD-MOFs and synthetic antioxidants (BHT, PG and Vc). Reprinted with permission [67]. Copyright 2023, Elsevier. Different letters on the lines indicate statistically significant difference at *p* ≤ 0.05 according to one-way ANOVA test.

Antioxidant activities of β-CD-MOFs encapsulated with clove essential oil (CEO) were found to be better compared to CEO and CEO/β-CD system (Figure 22). The superoxide radical scavenging activity of CEO was significantly improved after β-CD-MOFs inclusion and the increase in activities was 20.74% at 1 mg/mL. The hydroxyl radical scavenging activity of CEO was also improved after encapsulation in β-CD-MOFs and was 12.84% at 5 × 10^−5^ mg/mL [67]. The DPPH antioxidant assay also indicated similar results when (-)-epigallocatechin gallate (EGCG) was encapsulated inside γ-CD-MOFs [78]. The antioxidant activity of free EGCG drops rapidly from 55% to 11% after 5 days. In contrast, the EGCG protected by CD-MOF maintained its activity well in the 5 days. The antioxidant activity of CD-MOF-EGCG only dropped by 8% after 5 days.

### 7.7. Antibacterial Studies

**Figure 23:**
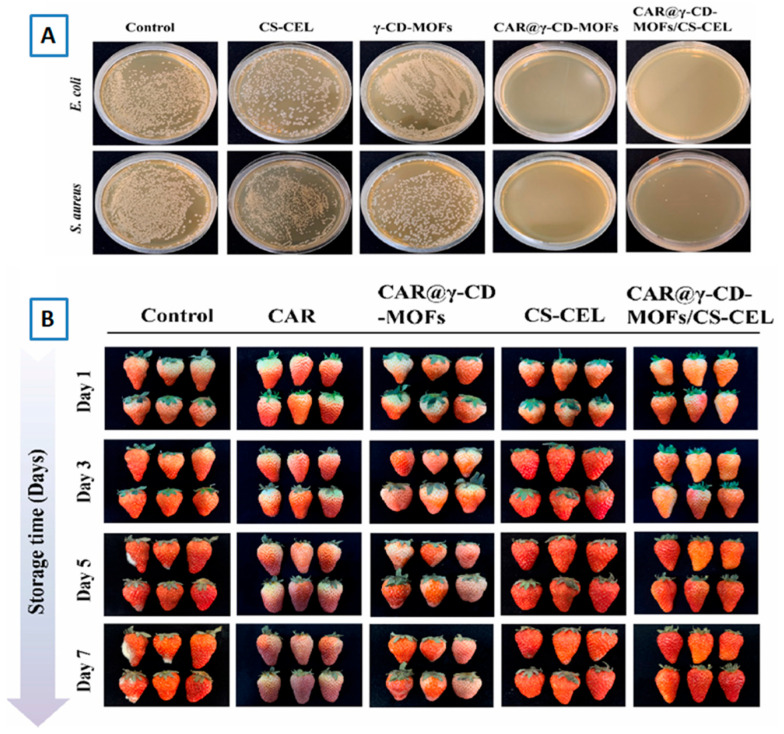
(**A**) Antibacterial activity of mechanism of carvacrol (CAR)-γ-CD-MOFs/Chitosan–Celluslose (CS-CEL) composite film. (**B**) Preservation effects of pure CAR, CAR@γ-CD-MOFs, CS-CEL, and CAR@γ-CD-MOFs/CS-CEL film on strawberries. Reprinted with permission [61]. Copyright 2024, Elsevier.

It is one of the most important tests to be carried out for the MOF inclusion complex that is intended to be used in food-based applications. The antibacterial activity of loaded MOF can be evaluated by calculating the Minimum Inhibitory Concentration (MIC) and Minimum Bactericidal Concentration (MBC). It was reported that without caffeic acid (CA), loading the γ-CD-MOF had no antibacterial effects but CA-CD-MOF showed MIC at 25 mg⋅mL^−1^ for *E. coli O157:H7* and *S. aureus.* The MBC for CA-γ-CD-MOF was 25 mg⋅mL^−1^ in *E. coli O157:H7* and no value could be observed in the measurement range in *S. aureus* [38]. In a different work, the colony-forming unit (CFU) method was used to evaluate the antibacterial activity of curcumin (Cur)-loaded γ-CD-MOFs. The results showed that initially, γ-CD-MOFs had almost no antibacterial effect but after the addition of curcumin, Cur-CD-MOFs showed excellent antibacterial properties in a time-dependent manner, and a lethality of 100% to *E. coli* and *S. aureus* at 4 h and 8 h [47] (see Figure 23).

### 7.8. Measurement of Other Properties Indicative of Food Preservation

Changes in properties such as hardness, weight loss, color change, etc. are all indicative of the degradation of the food product. These changes occur due to factors such as deterioration in cell structure, change in their respiratory patterns, destruction of cell walls, and growth of microbes. The study of these properties helps us to evaluate the effect of CD-MOF-IC applied to preserve the food under different conditions.

**Figure 24:**
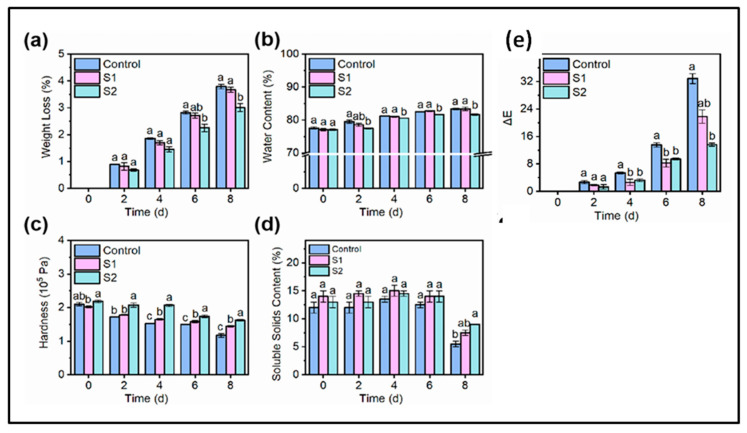
(**a**) Weight loss, (**b**) water content, (**c**) hardness, and (**d**) soluble solid content. (**e**) E (the total color difference) of emperor bananas treated with control group, S1 group (Gr-β-CD complex), and S2 group (GR-β-CD-MOF). Reprinted with permission [63]. Copyright 2024, American Chemical Society. Different letters above the bars indicate statistically significant difference at *p* < 0.05 according to one-way ANOVA.

In a study to understand the effect of thymol (THY) on the preservation of cherry tomatoes [43], it was found that after 15 days of storage γ-CD-MOFs showed the lowest decay index of 18.75% compared to the control (67.5%), α-CD-MOF-THY (33.3%), and β-CD-MOF-THY (48.75%). γ-CD-MOFs also showed the lowest weight loss of 5.17% after 15 days. However, the hardness of the cherry tomatoes was well maintained by α-CD-MOF-THY. Bai et al. [63] conducted a similar study and it was observed that the quality of the emperor banana became worse over a period of 8 days which could be observed through the reduction in weight and hardness of the bananas. However, the decrease in the hardness and weight was lower in the case of geraniol-encapsulated β-CD-MOFs (Gr-β-CD-MOFs) compared to geraniol just encapsulated in β-CD (Gr-β-CD) and the control group. After 8 days, the hardness of the control group decreased to 1.17 × 10^5^ Pa, Gr-β-CD decreased to 1.45 × 10^5^ Pa, and Gr-β-CD-MOF decreased to 1.62 × 10^5^ Pa. The water content increase which occurs due to degradation of both organic and inorganic matter was also found to be more in the control and Gr-β-CD compared to Gr-β-CD-MOFs, indicating the effect of geraniol on slowing the decay process of emperor bananas. The experimental result also demonstrates that GR-β-CD-MOF effectively retards the reduction in soluble solids and enhances the quality of emperor banana preservation during storage. A lower total color change was also observed when GR-β-CD-MOF was used for preservation (Figure 24)

### 7.9. Biocompatibilty and Cytotoxicity

The hemolysis assay was performed in order to evaluate hemocompatibility of geraniol included in β-CD-MOF. GR-β-CD-MOF group exhibited a pale red color like that of the saline group (negative control), while the TritonX-100 group (positive control) displayed a vivid red color, indicating a severe hemolytic reaction. Normalized to 100% absorbance of the Triton X-100 group, both the β-CD MOF and GR-β-CD-MOF groups demonstrated hemolysis values below 5%, which is within acceptable limits. These findings suggest that GR-β-CD-MOF possesses favorable hemocompatibility and can be safely administered, even if ingested into the body. The biocompatibility of GR-β-CD-MOF was investigated by using mouse embryonic fibroblasts (NIH 3T3) as the cellular model. The absorbance of the group with a concentration of GR-β-CD-MOF of 0 mg/mL was normalized to represent full cell viability (100%). Compared to the control group, no significant decrease in cell viability was observed for the groups with concentrations ranging from 0.25 to 1.0 mg/mL of GR-β-CD-MOF. However, when the concentration increased further to between 1.5 and 3.0 mg/mL, although there was a decline in cell survival rate, it remained above 50%. This decrease may be attributed to potential cytotoxicity associated with high concentrations of GR present in these samples; nevertheless, it is noteworthy that compared to the control group, GR-β CD-MOF exhibited a minimal impact on cell proliferation and demonstrated favorable biocompatibility [63].

**Figure 25:**
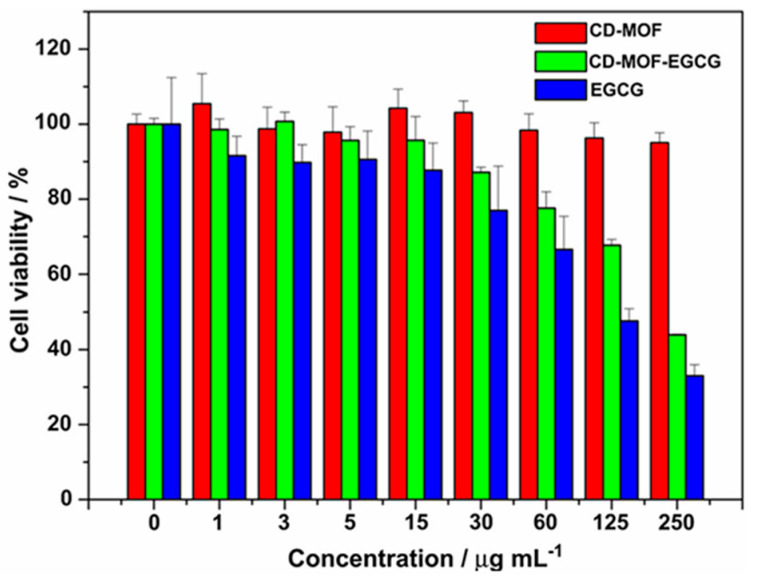
In vitro cell viabilities of C6 cells against the CD-MOF with and without EGCG loading at various concentrations. Reprinted with permission [78]. Copyright 2019, Springer Nature.

In vitro cell viabilities of CD-MOF, CD-MOF-EGCG, and free EGCG on C6 cells were evaluated by MTT assay to study the toxicity of CD-MOF and CD-MOF-EGCG. As shown in Figure 20, after an incubation time of 24 h with C6 cells, the C6 cells treated with the CD-MOF without loading EGCG exhibited a high cell viability (95%) when the concentrations of the CD-MOF ranged at 0–250 μg/mL^−1^, indicating that the synthesized CD-MOF nanocrystals were safe, biocompatible, and nontoxic. However, after loading with EGCG, the CD-MOF-EGCG nanocrystals showed a stronger cell growth inhibition effect on C6 cells compared to the presence of CD-MOF. The C6 cell viability value decreased to 44% when the concentration of the CD MOF-EGCG was only 250 μg/mL^−1^ (see Figure 25).

**Figure 26:**
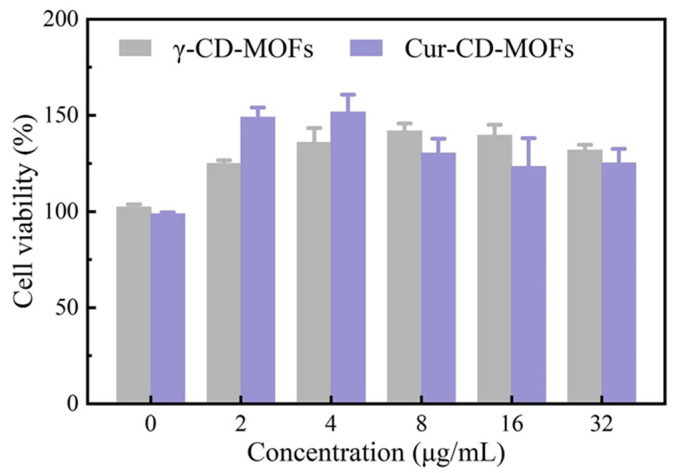
HepG2 cells’ survival percentage after incubation with different concentrations of γ-CD-MOFs and Cur-CD-MOFs for 12 h by CCK-8 Kit assay. Reprinted with permission [47]. Copyright 2023, Elsevier.

Considering the critical cytotoxicity of the preservative materials, human liver carcinoma cells (HepG2) were chosen to investigate the in vitro cytotoxicity and biocompatibility of γ-CD-MOFs and Cur-CD-MOFs using the CCK-8 Kit assay. Figure 26 shows that the viability of HepG2 cells is more significant than 100%, indicating that there was no cytotoxicity to HepG2 cells, which were then incubated with γ-CD-MOFs and Cur-CD-MOFs (0–32 μg/mL) materials for 12 h. These results indicated that microporous γ-CD-MOFs had good cytocompatibility as a potential curcumin carrier. When used as s fillers in food packaging, Cur-CD-MOFs pose a negligible toxicity risk [47].

**Figure 27:**
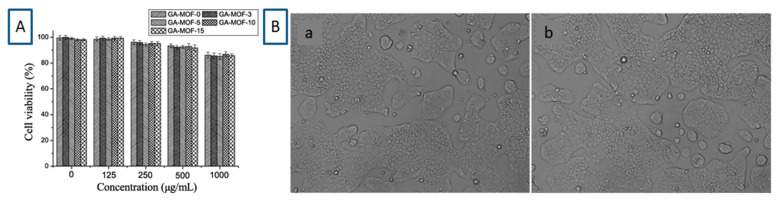
(**A**) In vitro cell viability of Caco-2 cells against the CD-MOF crystals prepared with different ultrasonic time. (**B**) Optical microscopy images of Caco-2 cells after being cultured for 24 h (**a**) control, (**b**) with GA-CD-MOFs at concentrations of 1000 μg/mL. Reprinted with permission [56]. Copyright 2019, Elsevier.

In a study by Qiu et al. [56], the cell viability of GA-loaded CD-MOFs was measured (Figure 27A) and CD-MOFs when present at low concentrations (<500 μg/mL) showed a high cell viability (>90%). Even at a high concentration (1000 μg/mL), the cell viability was still above 85%, indicating that the GA-loaded CD-MOFs were safe and nontoxic. The results from optical microscopy also supported the fact that the GA-CD-MOFs exhibited low toxicity in this cell model. The Caco-2 cells showed adherent growth ability (Figure 27B), indicating that they survived and grew well. No clear difference could be seen in the morphology of Caco-2 cells between cells incubated with CD-MOFs and control cells.

## 8. Conclusions and Future Trends

We firmly believe that the use of CD-MOF’s and their inclusion complexes for the development of biodegradable, biocompatible, non-toxic and cost efficient food preservation systems has multiple advantages. As we discussed CD-MOF’s have the capability to maximize the potential of bio-volatile compounds by increasing their stability and thus making it easier for their usage in various applications like medical, textiles and obviously food industry. Not only that CD=MOFs also possess a control release mechanism which help in increasing the shelf life of a food product for a longer period of time. The biodegradability and biocompatibility of CD-MOFs is another added advantage. The dual nature of CD-MOFs i.e., the ability to absorb as well as release is a boon for food preservation. As we observed that CD-MOF can also absorb ethylene leading to slow degradation process of a fruit or vegetable and at the same moment it can absorb and release ethylene for the rapid ripening of the natural food product. 

Even after several advantages associated CD-MOF usage, there are still areas which are needed to be explored. We reckon that there is a need of further studies especially concerning the toxicity effects of CD-MOFs. Using the CD-MOFs with different metal can induce toxicity which can be harmful even on a minimal scale. We observed that most of the studies involved the usage of K^+^ for the production of CD-MOFs and we think that there is a need to explore other biocompatible ions like Ca^2+^, Ti^4+^ etc. The usage of functionalized cyclodextrins for the formation of CD-MOFs or metal complexes should also be explored and the products should be used in various applications in food industries. There is need to focus more on the various techniques that can be used to develop CD-MOF based food preservation systems. For example, developments of packaging films including CD-MOF inclusion complexes should be upgraded to industrial scale and techniques like extrusion or blow molding should be modified and tried for this purpose. Another domain that requires more attention is the usage of different techniques for CD-MOF synthesis. We discussed that Vapor diffusion and Ultrasonication are mostly used for the development of CD-MOFs, but other techniques like microwave assisted should be also investigated in deep. There is a need for green and cost effective techniques that can help produce MOFs on an industrial scale. Ultimately, there are immense possibilities of CD-MOFs to be used in the food application which can lead to a reduction in food waste, maintaining nutritive nature of food product for a longer period of time for human consumption thus contributing to different sustainable goals set up by the European Union.

## Figures and Tables

**Table 1:** Properties of the native cyclodextrins.

Properties	α-CD	β-CD	γ-CD
Number of glucose units	6	7	8
Molecular weight (g/mol)	972	1135	1297
Solubility in water at 25 °C (%, *w*/*v*)	14.5	1.9	23.2
Melting point (°C)	275	280	275
Cavity diameter (Å)	4.7–5.3	6.0–6.5	7.5–8.3
External diameter (Å)	14.6	15.4	17.5
Crystal forms (from water)	Hexagonal plates	Monoclinic parallelograms	Quadratic prisms
European trade name as food additives	E-457	E-459	E-458

**Table 3:** Comparison of K-γ-CD-MOFs with different counter ions [52,60].

	KOH-γ-CD-MOF	KCl-γ-CD-MOF	KAc-γ-CD-MOF	KBz-γ-CD-MOF
Formula	C_48_H_124_K_2_O_64_	C_92_H_212_KClO_86_	C_302_H_637_K_7_O_222_	C_110_H_172_K_4_O_86_
Space group	I432	P4212	R32	-
Formula weight	1803.66	2769.16	8110.78	3026.88
Crystallite structure	Cubic	Tetragonal	Trigonal	Trigonal
Cavity size	0.3–1.0 nm	0.2–2.5 nm	0.2–1.0 nm	0.40 ± 0.40 nm

**Table 4:** Solubilities of ALPs, ALP-β-CD, and ALP-β-CD-MOFs in various solvents [41]. * Solubility (g/100 g solvent).

Solvent	ALP	ALP/β-CD	ALP-β-CD-MOFs
Water	1.15 ± 0.12 *	1.87 ± 0.24 *	11.93 ± 0.43 *
Methanol	0.43 ± 0.04 *	0.46 ± 0.09 *	0.56 ± 0.12 *
Ethanol	0.22 ± 0.07 *	0.29 ± 0.06 *	0.40 ± 0.05 *
